# COVID-19 evolution during the pandemic – Implications of new SARS-CoV-2 variants on disease control and public health policies

**DOI:** 10.1080/21505594.2021.1877066

**Published:** 2021-01-25

**Authors:** Cock van Oosterhout, Neil Hall, Hinh Ly, Kevin M. Tyler

**Affiliations:** a School of Environmental Sciences, Norwich Research Park, University of East Anglia, United Kingdom; b Earlham Institute, Norwich Research Park, Norwich, United Kingdom; c Center of Excellence for Bionanoscience Research, King Abdul Aziz University, Jeddah, Saudi Arabia; d Department of Veterinary & Biomedical Sciences, University of Minnesota Twin Cities, St. Paul, MN, United States; e Norwich Medical School, University of East Anglia, Norwich, United Kingdom

In recent weeks, several new strains of SARS-CoV-2, the causative agent of COVID-19, have emerged. These variants have evolved an increased transmission rate compared to the original strains, which makes controlling this virus even more challenging. What has happened, and how do we best respond now?

One strain – UK B.1.1.7 lineage (variant of concern 202012/1) in particular is currently sweeping the UK. A recent report has suggested that this strain has a significantly higher potential rate of transmission (R_0_) compared to previous variants [1,2]. Genomic analysis of the strain found substantial novel sequence variation caused by mutations, which may provide a biological reason for the observed increase in transmissibility. Initial assessments suggest the novel variants show an improved interaction with host cell receptors, such as ACE2 on epithelial cells [3]. This enables the virus to better establish and propagate infections, resulting in higher levels of virus in the host and increased rate of transmission [4,5].

A recent analysis of the transmission characteristics of the B.1.1.7 variant stratified by age suggests that its higher R_0_ may be largely attributable to an increase in transmission to and among school-age children, whilst infection rates for older people appear to be less affected [2]. The successful emergence of such variants is most rapid with a higher R_0_. Such so-called “selective sweeps” are common in pathogen evolution. According to the Red Queen hypothesis [6], each increment in the fitness of the pathogen results in an equivalent reduction in fitness of the host. If the R_0_ of the most virulent variant can be kept below one, it will not be able to further establish itself in the host population and replace the original strain. In simple terms, containing the outbreaks of highly virulent strains now won’t just save lives in the next months, but it will also save many lives in the years to come. Thus, a policy of minimizing the R_0_ by closing schools will help to contain the establishment of highly virulent strains. Employing vaccines is a more long-term strategy, but it will take several months to become an effective control measure.

Governments are negotiating a precarious balance between saving the economy and preventing COVID-19 fatalities. However, the roll-out of economic stimulus packages and related activities in many countries appears to have fuelled the rate of person-to-person transmission. This created two distinct problems. Firstly, at the start of the winter, the population number of the virus continued from a much higher base than would otherwise have been the case. With an exponentially growing rate of the infection (R_0_ > 1), the time it takes to increase the number of infected hosts from N to 2 N people is the same, irrespective of N. In other words, if we had halved the number of infections, we would have had (approximately) half the number of cases now. Secondly, the probability that the pathogen evolves, and that the next infection is caused by the mutant strain of the virus, is equal to the mutation rate for each transmission (µ) [7]. By not absolutely minimizing the R_0_ when we had the chance, we extended the pathogen transmission chains, allowing it to mutate and evolve into more virulent variants. Put simply, more transmission leads to a higher chance of evolution of new strains, and it promotes selective sweeps and the establishment of such strains in the susceptible population. As such, continuing public health efforts to encourage vaccination as well as continued use of proper personal protective equipment (PPE), such as proper masking and maintaining safe social interactions, is of utmost importance.

COVID-19 vaccine deployment is now underway, but a threat to vaccine effectiveness comes from other emergent strains, both existing and yet to come. For example, another highly virulent SARS-CoV-2 variant has been identified in South Africa (SouthAfricaV501.V2 clade), which like B.1.1.7, appears to be transmitting more quickly than other strains. This lineage has rapidly become the dominant circulating strain, and it too is mutated in several areas of the viral spike protein [3] as are a group of Brazilian (B 1.1.28) variants now predominating in Amazonas state [8]. The fear is that variation generated by mutation could give rise to vaccine-resistant strains in the long term. Such vaccine escaped mutants can potentially be favoured during protracted infections in patients with a weakened immune response and longer transmission chains. Such conditions increase the input of new mutations and the time for natural selection to act on this novel variation. Furthermore, rather than selecting for increased transmissibility, the next stage of virus evolution may involve adaptations that increase the duration that a host is contagious. After all, in directly transmitted parasites, R_0_ is the product of transmission efficiency (β), the contact rate between susceptible hosts (*c*) and the duration (*d*) that an infected host is contagious [9]. A sufficient level of vaccination coverage will reduce the number of contacts between susceptible hosts, and hence, selection pressures for increased R_0_ are likely to involve adaptations that prolong the infection. Finally, strains evolving independently in reservoir hosts (e.g. mink) have also been shown to contain viral spike protein mutations and be less readily neutralized by immune serum [10]. Continued virus evolution in reservoir animal host, followed by spillback events into susceptible human hosts, poses a significant long-term risk to public health. SARS-CoV-2 can infect a wide range of host species, including cats, dogs, mink, and other wild and domesticated species [11], and hence, the vaccination of domesticated animals might be required to halt further virus evolution and spillback events.

Whilst the vaccination campaigns against SARS-CoV-2/COVID-19 are being rolled out worldwide, new virus variants are likely to continue to evolve that have the potential to sweep through the human population. Higher transmission rates require a higher level of immunity to bring R_0_ below one [12] and a more transmissible virus strain, such as the UK B.1.1.7 requires more people to be vaccinated in order to keep the virus and the disease it causes under control. Furthermore, higher transmission rates increase the evolutionary potential of the virus by increasing the input of new mutations, potentially resulting in even more virulent strains. Vaccination against a viral pathogen with such high prevalence globally is without precedent and we, therefore, have found ourselves in unchartered waters. However, what we can be certain about is that, as long as vaccines remain effective, a higher uptake of the vaccines will: (1) reduce the number of COVID-19-related deaths, (2) stem the spread of the transmissible strain of the virus, and (3) reduce risk of the evolution of other, even more, virulent strains in the future. Furthermore, it is not unthinkable that vaccination of some domesticated animal species might also be necessary to curb the spread of the infection.

Humanity is faced with a new reality. The faster we adapt, the better our long-term prospects. We must stop the evolution and spread of more virulent virus strains now. We, therefore, support public health policies with strict control measures in order to protect our public health system, our individual wellbeing, and our future.
